# Cardiovascular effects of *Helichrysum ceres* S Moore [Asteraceae] ethanolic leaf extract in some experimental animal paradigms

**Published:** 2008-10

**Authors:** Cephas T Musabayane, Dave R Kamadyaapa, Mavuto Gondwe, Kogi Moodley, John AO Ojewole

**Affiliations:** Department of Human Physiology, School of Medical Sciences, University of KwaZulu-Natal, Durban; Department of Human Physiology, School of Medical Sciences, University of KwaZulu-Natal, Durban; Department of Human Physiology, School of Medical Sciences, University of KwaZulu-Natal, Durban; Department of Human Physiology, School of Medical Sciences, University of KwaZulu-Natal, Durban; Department of Pharmacology, School of Pharmacology Sciences, University of KwaZulu-Natal, Durban

## Abstract

**Summary:**

The aim of this study was to examine some in vivo and *in vitro* cardiovascular effects of *Helichrysum ceres* leaf ethanolic extract (HCE) in experimental animal paradigms. The acute effects of HCE on blood pressure were studied in anaesthetised normotensive male Wistar rats challenged with intravenous hypotonic saline infusion after a 3.5-hour equilibration for four hours of one-hour control, 1.5-hour treatment and 1.5-hour recovery periods. HCE was added to the infusate during the treatment period. Sub-chronic hypotensive effects of HCE were examined in weanling Dahl salt-sensitive (DSS) genetically hypertensive rats, which progressively develop hypertension with age, treated with HCE (80 mg/kg) every third consecutive day for seven weeks. Isolated atrial muscle strips, portal veins and descending thoracic aortic rings of healthy normotensive Wistar rats were used to investigate the vascular effects of HCE.

Acute HCE administration caused a significant (*p* < 0.05) fall in blood pressure in the normotensive anaesthetised Wistar rats. DSS hypertensive rats treated with HCE displayed low arterial blood pressure and heart rate values from weeks five to seven. HCE produced concentration-dependent negative inotropic and chronotropic effects on rat isolated electrically driven left, and spontaneously beating right atrial muscle preparations, respectively. HCE also evoked concentration-dependent relaxation responses of endothelium-intact aortic rings and portal veins isolated from healthy normotensive Wistar rats. The vasorelaxant effects of HCE in intact aortic rings were significantly reduced, but not completely abolished by adding endothelial-derived factor (EDRF) inhibitor, L-NAME, suggesting that the vasorelaxant effect of the extract is mediated via EDRF-dependent and independent mechanisms.

The results of the study suggest that the hypotensive action of HCE is elicited, in part, directly by decreasing myocardial contractile performance and total peripheral vascular resistance due to its negative inotropic and chronotropic effects on rat isolated atrial muscle strips; and vasorelaxant effects on isolated vascular smooth muscles. The observed cardiovascular effects of HCE partly support the basis for its use in the management of high blood pressure in folkloric medicine.

## Summary

Prevailing economic conditions and poor health services in many developing populations necessitate the development of alternative low-cost therapies to manage various human ailments. Plant extracts present another option of easily accessible sources of new drugs. Indeed, some plant extracts have been reported to be effective in the management of hypertension, including complications such as coronary heart disease, angina, arrhythmias and congestive heart failure.^1^-^3^ Furthermore, pharmaceutical cardiac glycoside preparations have been formulated from plant extracts.^4^

Ethnomedical evidence indicates that extracts of the genus *Helichrysum* [family, Asteraceae] possess anti-inflammatory and anti-allergic properties.^5^ Traditional folklore medicinal healers in southern Africa have used *Helichrysum ceres* S Moore [Asteraceae] to treat kidney and cardiorespiratory disorders.^6^ Recent laboratory studies suggest that the hypotensive effects of *H ceres* leaf extract in anaesthetised male Sprague-Dawley rats can, in part, be attributed to the extract’s natriuretic and diuretic properties.^7^ On this basis, we speculated that *H ceres* leaf extract may avert the progression of high blood pressure in the Dahl salt-sensitive rats (DSS), a genetic model of salt-sensitivity hypertension.^8^ DSS rats progressively develop hypertension when fed on diets with normal salt concentrations, but become extremely hypertensive on a high-sodium diet.^9^

The main aim of the present study was, therefore, to assess the effects of H ceres leaf extract on mean arterial blood pressure in normotensive Wistar and hypertensive DSS rats. We also evaluated the effects of the leaf extract on the myocardial contractile performance of rat isolated atrial muscle strips, and the vasodilatory effects on isolated portal veins and thoracic aortic rings of healthy normal Wistar rats. We envisaged that establishment of the mechanism(s) of the cardiovascular effects of the leaf extract would provide scientific evidence for the development of an inexpensive and accessible source of novel drugs for the treatment of hypertension and certain cardiac disorders in underprivileged, developing populations.

## Materials and methods

Methoxamine hydrochloride (ME), acetylcholine chloride (ACh), indomethacin, NG-nitro-L-arginine-methyl-ester (LNAME), methylene blue, atropine sulphate (ATR), glibenclamide, (±)-propranolol hydrochloride, (-)-noradrenaline hydrochloride (NA), and nifedipine were obtained from Sigma (St Louis, MO, USA). All other chemicals were of the analytical grade and supplied by Merck chemicals (Pty) Ltd, (Wadenville, Germiston, South Africa). Indomethacin was dissolved in 0.5% sodium bicarbonate. Glibenclamide was dissolved in dimethyl sulphoxide (DMSO). All other drugs, including Krebs’ solution were freshly prepared in deionised water.

Leaves from *Helichrysum ceres* (S Moore) [Asteraceae] identified by Professor H Baijnath, the former chief taxonomist/curator of the University of KwaZulu-Natal’s Department of Botany, were collected around Durban, South Africa, between January and June 2005. A voucher specimen of the plant has been deposited in the University’s Botany Herbarium.

Ethanolic *H ceres* leaf extract was prepared as previously described.^10^ One kilogram of fresh leaves was air-dried at room temperature, cut into small pieces and ground into powder using a commercial Waring blender. The powdered leaves were soaked in 99% ethanol (2.5 l) at room temperature for 48 hours, with occasional shaking, and extracted twice. The combined ethanol extracts were filtered using 30-cm filter paper (Whatman, England) and concentrated to dryness using a rotary evaporator at 55ºC (Buchi, Lotavapor, Essen, Germany). Freeze-drying and solvent elimination under reduced pressure produced 22.75 g of a light-brown, powdery extract (HCE), a yield of 2.28%. The crude extract was used without further purification. Portions of the extract were weighed and dissolved in deionised water for use on each day of our experiments.

Male Wistar (250–300 g) and weanling Dahl salt-sensitive rats (100–150 g), bred and housed at the Biomedical Research Unit, University of KwaZulu-Natal were used in this study. The rats were maintained on a 12-h light/12-h dark regime, and given both food (Epol diet 4700, Epol, South Africa) and water *ad libitum*. Procedures involving animals and their care were conducted in conformity with institutional guidelines of the University of KwaZulu-Natal.

The acute and sub-chronic effects of HCE on mean arterial blood pressure (MAP) were examined *in vivo* in the Wistar and DSS rats, respectively (DSS rats develop hypertension as they age). The effect of HCE on myocardial contractile performance was evaluated on isolated rat atrial muscle strips, while the vasodilatory effects of the extract were examined on isolated thoracic aortic rings and portal veins of healthy Wistar rats.

## Acute *in vivo* effects of HCE on MAP and heart rate

Wistar normotensive rats were prepared for acute blood pressure studies using a method previously described in detail.^10^ The animals were anaesthetised by an intraperitoneal injection (0.11 g/kg body weight) of inactin (5-ethyl-5-(1′-methylpropyl)-2-thiobarbiturate (Sigma Aldrich, St Louis, Missouri, USA) and transferred to a heated table (CF Palmer, London, England) which maintained the body temperature at 37 ± 1°C throughout the experiment, monitored by a rectal probe. Each rat was tracheotomised to maintain a clear airway. Cannulae were inserted into the jugular vein for intravenous infusion of 0.077 M NaCl at 9 ml/h (Harvard syringe infusion pump 22, Harvard Apparatus, Holliston, Massachusetts, USA).

A heparinised cannula inserted into the carotid artery directly measured blood pressure and heart rate at 30-min intervals via a pressure transducer (Statham MLT 0380, Ad Instruments, Bella Vista NSW, Australia) compatible with the PowerLab System ML410/W (Bella Vista, NSW, Australia). The urinary bladder of each rat was also cannulated, for urine voidance, with a similar calibre polythene tubing via an incision in the abdominal wall.

Following a 3- to 5-hour equilibration period, measurements were recorded over the four-hour post-equilibration period of one-hour control, 1.5-hour treatment and 1.5-hour recovery periods. In those animals in which the effects of the extract were studied, HCE was added to the infusate at 360 μg/h for 1.5 hours (treatment period), resulting in a total dose of 1.8 mg/kg (for a 300-g rat), before the animals were returned to infusate alone for the last 1.5 hours (recovery period). The depth of anaesthesia was monitored throughout the experiment and additional intravenous bolus doses of inactin (0.05 g/kg body weight) were administered when necessary. The administration rates of the extracts were chosen from previous experience.^7^

## Chronic *in vivo* effects of HCE on MAP

In order to evaluate the long-term effects of HCE on blood pressure, separate groups comprising control and extract-treated groups of DSS rats (*n* = 8, per group) were used. Mean arterial blood pressure was measured every third consecutive day for seven weeks at 09:00 in both the untreated control group and the HCE-treated (120 mg/kg, po) DSS rats. Control rats were similarly treated with deionised water (3 ml/kg). A tail-cuff computerised blood pressure monitor (IITC Model 31 computerised blood pressure monitor, Life Sciences, Woodland Hills, CA) was used to assess the cardiovascular effects of HCE. The method was standardised and used routinely in our laboratory and is described elsewhere in detail.^10^

## *In vivo* inotropic and chronotropic effects of HCE

The inotropic and chronotropic effects of HCE on myocardial contractile performance were evaluated on rat isolated atrial muscle strips of normotensive Wistar rats, as previously described.^10^

Isolated rat atria were suspended under 1.0-g tension in a 30-ml Ugo Basile organ bath (Ugo-Basile, Comerio, Italy) containing Krebs-Henseleit solution (KHS) of the following composition in mM: NaCl, 118; KCl, 4.7; NaH_2_PO_4_.2H_2_O, 1.28; NaHCO_3_, 25.0; CaCl_2_.2H_2_O, 2.52; MgCl_2_, 1.2; glucose, 5.55. The atrial muscle preparations were left to equilibrate for 45–60 minutes, during which time the physiological bathing solution was changed every 15 minutes. The organ bath was maintained at 36 ± 1°C and continuously gassed with 95% O_2_ and 5% CO_2_.

To evaluate the contractile force of the extract (inotropic), the electrically driven left atrium was impaled on a thin platinum wire electrode and stimulated (5–10 mV) with square wave pulses of 5-ms duration at a frequency of 3 Hz via an SRI stimulator (Preamplifier, Bioscience, UK). To examine the extract’s effect on atrial pacemaker activity (chronotropic), spontaneously beating right atria were set up under the same physiological experimental conditions. Two isolated electrically driven left atrial muscle strips, and two isolated spontaneously beating right atrial muscle preparations were always set up at a time (one used as the test, and the other as the control) to allow for changes in the atrial muscle sensitivity.

Concentration–response curves for HCE (0–160 mg/ml) and/or reference agonist drugs were obtained. Control atrial muscle strips were treated with volumes of deionised water equivalent to the volumes of bath-applied HCE solution (0.1–0.6 ml). The electrically provoked and spontaneous contractions of the atrial muscles, as well as the HCE (and reference agonist drug)-induced responses of the atrial muscle preparations were recorded isometrically by means of Ugo Basile force–displacement transducers and pen-writing Gemini recorders (model 7070). The effects of HCE/drugs were expressed as a percentage of the baseline values (*n* = 8 preparations for each concentration).

## Effects of HCE on isolated aortic rings

The *in vitro* vasodilatory effects of HCE on smooth muscles were evaluated using thoracic aortic rings and spontaneous myogenic contracting portal vein preparations isolated from normotensive Wistar rats.^10^

Aorta pieces (4–5 mm long) suspended under a resting tension of 1 g in a 30-ml Ugo-Basile organ bath containing KHS were left to equilibrate for 45–60 minutes. In some experiments, the endothelium was denuded mechanically *in situ* by gently rubbing the luminal surface of the aortic rings with moistened cotton wool. Control aortic rings with and without functional endothelium were pre-contracted with a single sub-maximal concentration of methoxamine hydrochloride (ME, 10 μM). Satisfactory and functional endothelium was checked by at least 10% and 70% relaxation, respectively, to 10^-6^ M ACh. After a sustained stable tonic contraction was achieved, concentration–response curves for HCE (0–160 mg/ml) and/or reference agonist drugs were obtained.

The involvement of endothelium-derived factors in HCE-induced relaxation was examined in intact aortic rings pre-treated with appropriate antagonists (N^G^-nitro-L-arginine methyl ester, L-NAME, 100 μM, a nitric oxide synthase inhibitor; methylene blue, 10 μM, a guanylate cyclase inhibitor; and indomethacin, 10 μM, a non-selective cyclooxygenase inhibitor).

To assess the role of potassium or calcium in the vasorelaxant effect of the extract, concentration–response curves of HCE were constructed in endothelium-intact aortic rings pre-contracted with potassium (K^+^, 20 mM) in the presence glibenclamide, and high potassium (K^+^, 80 mM), respectively, as previously described.^11^-^14^

The contractile and/or relaxant effects of all the reference drugs used, as well as HCE-induced relaxations of the isolated aortic ring preparations were recorded isometrically by means of Ugo Basile force–displacement transducers and pen-writing Gemini recorders.

## Effects of HCE on isolated portal veins

Each isolated rat portal vein preparation (2 cm long) suspended under a resting tension of 0.5 g in a 30-ml Ugo-Basile organ bath containing KHS was allowed to equilibrate for 60 minutes, following which, graded concentrations of HCE (10, 40 or 160 mg/ml) were added to the organ bath in separate preparations to establish its effects on the amplitude of myogenic contractions.

To investigate whether the effects of HCE were mediated through modulation of alpha-1 adrenergic receptors or voltage-operated calcium channels, some of the portal vein preparations were pre-treated with an alpha-1 adrenergic receptor blocker, prazosin (1 μM), and an L-type voltage-operated calcium channel blocker, nifedipine (1 μM), respectively, five minutes before re-establishing the cumulative concentration–dependent responses. Control venous muscle strips were treated with deionised water equivalent to the volumes of bath-applied HCE solution. Two isolated venous tissue preparations (one control and the other HCE- or reference drug-treated test) were always set up in order to make allowances for changes in the venous tissue sensitivity.

The HCE- and/or drug-induced responses of the venous smooth muscle preparations recorded isometrically by means of Ugo Basile force–displacement transducers and pen-writing Gemini recorders were calculated as a percentage of the baseline values (*n* = 8 preparations for each concentration).

## Statistical analysis

All results are expressed throughout as means ± SEM. Data were analysed by one-way analysis of variances (ANOVA) followed by Dunnett multiple-comparison tests. A value of *p* < 0.05 was considered significant. All statistical calculations were performed with GraphPad InStat Software (version 3.00, GraphPad Software, Inc, San Diego, California, USA).

## Results

## In vivo effects of HCE on MAP and heart rate

**Fig. 1.**
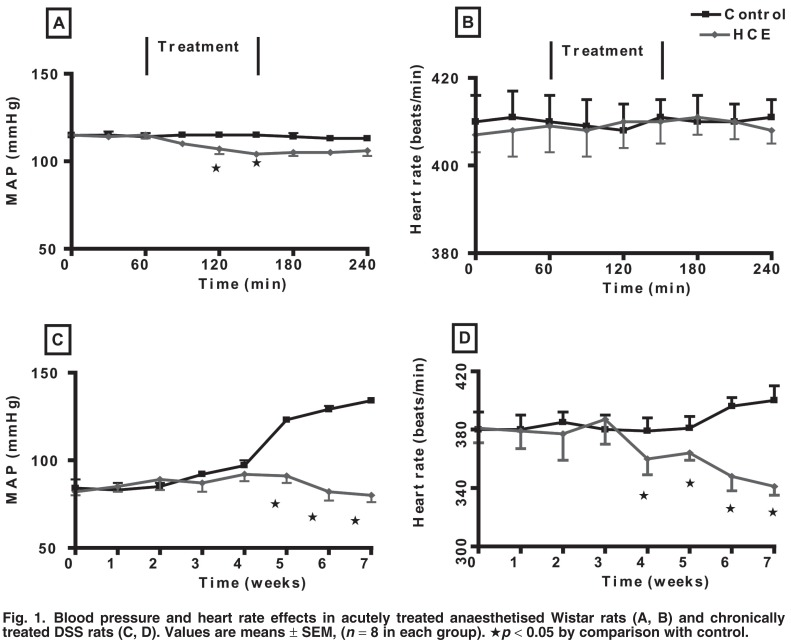
Blood pressure and heart rate effects in acutely treated anaesthetised Wistar rats (A, B) and chronically treated DSS rats (C, D). Values are means ± SEM, (*n* = 8 in each group). ★*p* < 0.05 by comparison with control.

Fig. 1 shows the acute and subchronic effects of HCE on mean arterial blood pressure and heart rate examined *in vivo* in normotensive Wistar and DSS hypertensive rats. The MAP and heart rate of Wistar rats challenged with continuous jugular infusion of 0.077 M NaCl at 9 ml/h did not change during the four-hour experiment with HCE (Fig. 1A, B). However, it caused a significant fall in the MAP during the 1.5-hour treatment period. The decrease persisted during the recovery period and was significantly lower at the end of the recovery period when compared to values recorded during the pre-treatment period and in control animals at the corresponding time. HCE had no significant effect on heart rate (Fig. 1B).

To evaluate the long-term effects of HCE on mean arterial blood pressure and heart rate, DSS rats were treated every third consecutive day for seven weeks at 09:00 with HCE (80 mg/kg, po). By comparison with values in week one and untreated animals after four weeks, HCE treatment of DSS rats prevented the expected age-related increase in blood pressure and slightly reduced the heart rate (Fig. 1C, D).

## In vitro inotropic and chronotropic effects of HCE

**Fig. 2.**
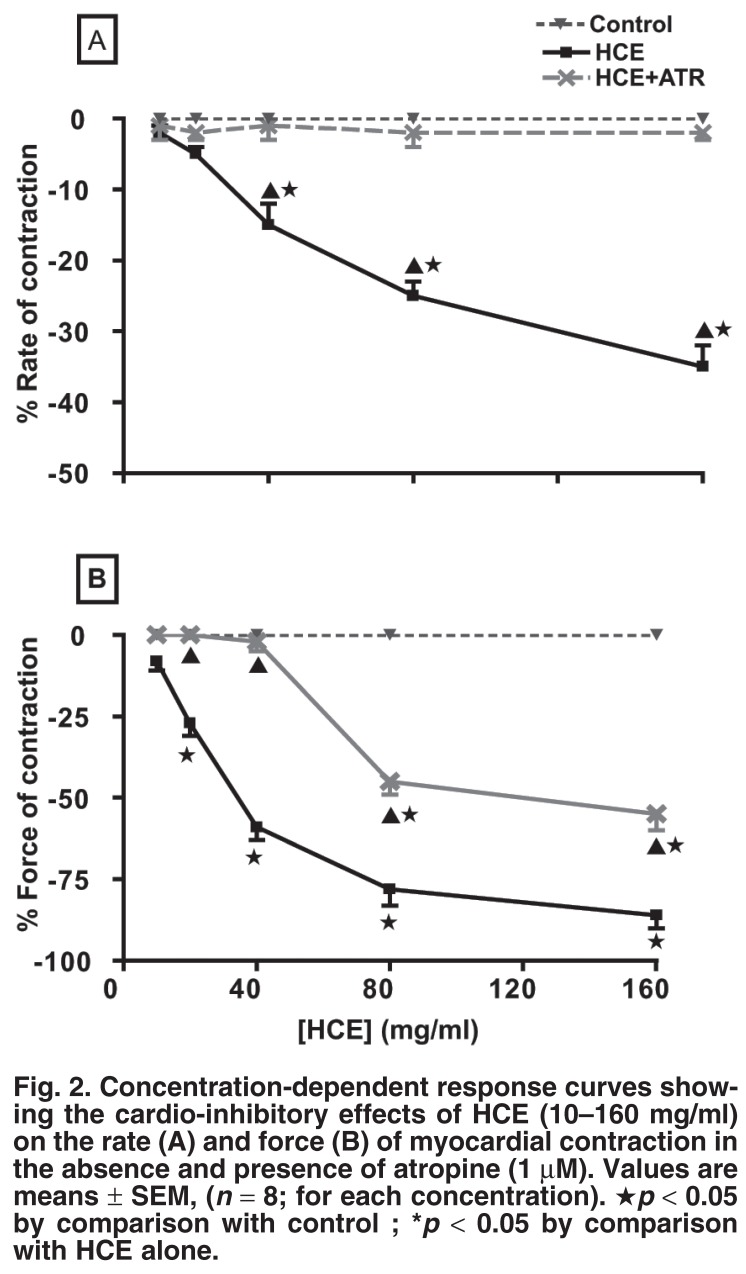
Concentration-dependent response curves showing the cardio-inhibitory effects of HCE (10–160 mg/ml) on the rate (A) and force (B) of myocardial contraction in the absence and presence of atropine (1 μM). Values are means ± SEM, (*n* = 8; for each concentration). ★*p* < 0.05 by comparison with control ; *p < 0.05 by comparison with HCE alone.

Fig. 2 shows the chronotropic and inotropic effects of HCE treatment on rat isolated spontaneously beating right, and electrically driven left atrial muscle strips, respectively. HCE reduced the spontaneous beating of the right atrial muscle preparations (negative chronotropy) in a concentration-dependent manner (Fig. 2A). To assess whether the negative chronotropic effect was mediated through activation of cholinergic receptors, the right atrial muscle preparations were pre-treated with atropine sulphate (ATR, 1 μM), a cholinergic receptor antagonist.

Pre-treatment of the atrial preparations with ATR elicited a complete abolition of the negative chronotropic effect of HCE (Fig. 2A). HCE significantly (*p* < 0.05) and concentration-dependently decreased the force of myocardial contraction (negative inotropy) of the rat isolated electrically driven left atria (Fig. 2B). Pre-treatment of the atrial preparations with atropine sulphate completely blocked the negative inotropic effects of lower concentrations of HCE, but the effects of higher concentrations of HCE were not completely abolished (Fig. 2B).

**Fig. 3.**
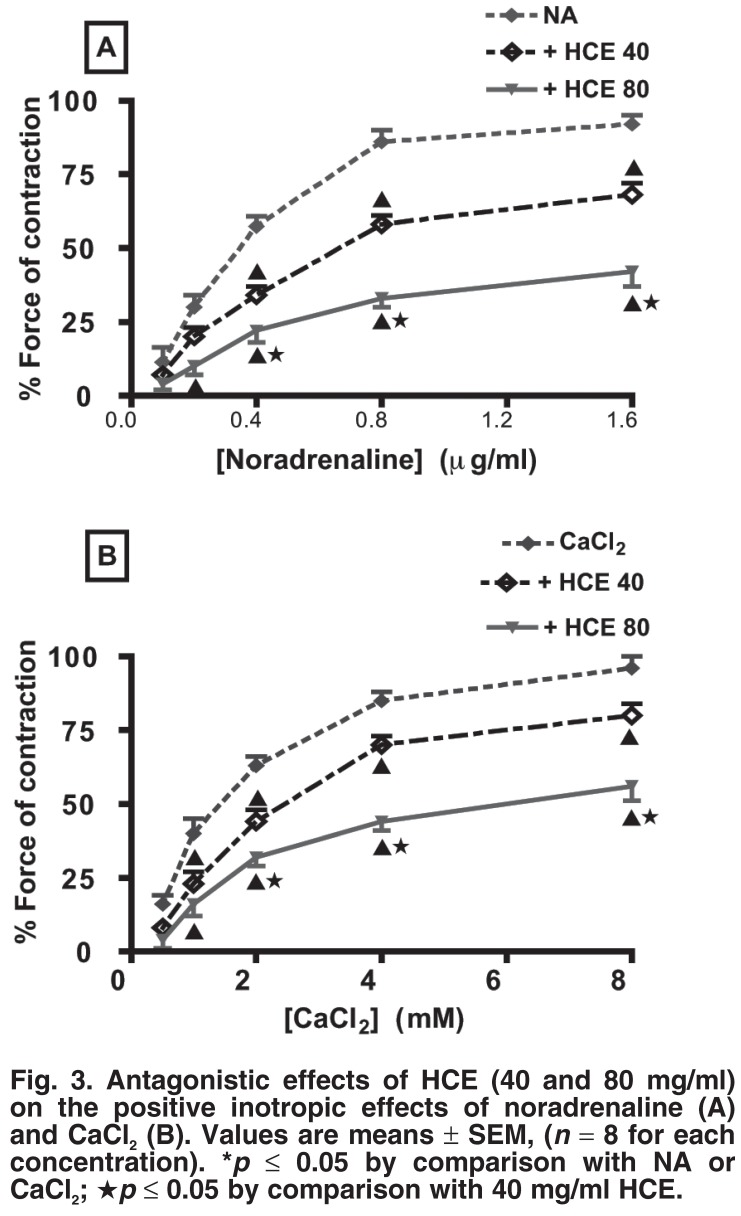
Antagonistic effects of HCE (40 and 80 mg/ml) on the positive inotropic effects of noradrenaline (A) and CaCl_2_ (B). Values are means ± SEM, (*n* = 8 for each concentration). *p ≤ 0.05 by comparison with NA or CaCl_2_; ★*p* ≤ 0.05 by comparison with 40 mg/ml HCE.

To determine whether HCE influenced the noradrenaline- or calcium-induced inotropic effects, cumulative concentration–response relationships for noradrenaline (0–1.5 ml) or CaCl_2_ (0–8 mM) were established in isolated electrically driven left atria in the absence or presence of HCE (40 or 80 mg/ml). Both noradrenaline and calcium induced concentration–response curves (positive inotropy) (Fig. 3A). Pre-incubation of aortic rings with HCE at 40 or 80 mg/ml significantly antagonised the noradrenaline- or calcium-induced positive inotropic effect in a concentration-dependent manner (Fig. 3B).

## Vascular effects of HCE in isolated aortic rings

**Fig. 4.**
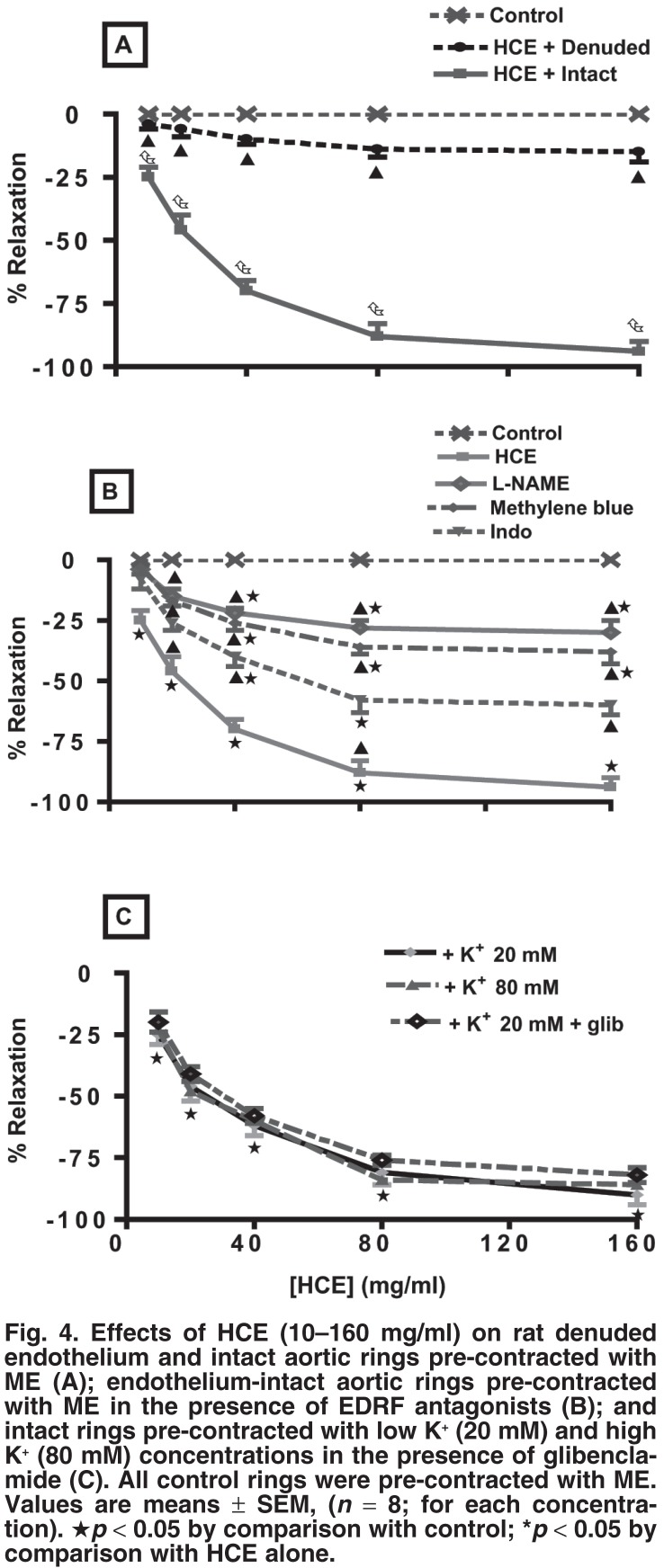
Effects of HCE (10–160 mg/ml) on rat denuded endothelium and intact aortic rings pre-contracted with ME (A); endothelium-intact aortic rings pre-contracted with ME in the presence of EDRF antagonists (B); and intact rings pre-contracted with low K^+^ (20 mM) and high K^+^ (80 mM) concentrations in the presence of glibenclamide (C). All control rings were pre-contracted with ME. Values are means ± SEM, (*n* = 8; for each concentration). ★*p* < 0.05 by comparison with control; *p < 0.05 by comparison with HCE alone.

Addition of cumulative concentrations of HCE (0–160 mg/ml) to aortic rings pre-contracted with 10 μM ME (α_1_-adrenoceptor agonist) evoked concentration-dependent relaxation responses of endothelium-intact aortic rings (Fig. 4A). The responses were significantly inhibited, but not completely abolished in endothelium-denuded aortic rings (Fig. 4A). The endothelium-intact aortic ring preparation was therefore used to study the roles of endothelial-derived vasodilators, calcium and potassium channels in the vasorelaxant effects of HCE.

In order to analyse the involvement of endothelial-related factors in the vasorelaxant effects of HCE, concentration–response curves of HCE (0–160 mg/ml) were constructed in separate preparations of ME pre-contracted endothelium-intact aortic rings in the absence or presence of EDRF inhibitors: nitric oxide synthase inhibitor (L-NAME), guanylate cyclase inhibitor, (methylene blue) and cyclooxygenase inhibitor, (indomethacin). The vasorelaxant effects of HCE were significantly reduced by the antagonists (Fig. 4B). Taken together, the above results showed that the vasorelaxant effect of HCE was, in part, endothelium-dependent.

Concentration–response curves of HCE (0–160 mg/ml) were established in endothelium-intact aortic rings pre-contracted with a low (K^+^, 20 mM) or a high potassium concentration (K^+^, 80 mM) to evaluate the role of potassium and calcium channels, respectively, on the vasorelaxant effects of the extract. In order to assess the involvement of KATP-sensitive channels, the rings were pre-treated with 3 μM glibenclamide (ATP-sensitive potassium channel blocker) before pre-contracting with K^+^ (20 mM). Vasorelaxation of aortic ring preparations pre-contracted with low potassium (K^+^ < 30 mM) concentration occurs via opening of the potassium channels.^15^,^16^ HCE induced a significant and concentration-dependent vasorelaxation in the endotheliumintact aortic rings pre-contracted with low potassium concentrations (Fig. 4C). Pre-treatment of the aortic rings with glibenclamide before pre-contracting with low K^+^ did not cause any change in the vasorelaxant effects of HCE (Fig. 4C).

To evaluate the role of calcium channels in the vasorelaxant effects of HCE, endothelium-intact aortic rings were precontracted with high concentrations of potassium (K^+^, 80 mM). HCE (0–160 mg/ml) induced a significant vasorelaxation in the endothelium-intact aortic rings pre-contracted with high K^+^ (Fig. 4C). Vasorelaxation in aortic preparations pre-contracted with high concentrations of potassium is known to be mediated by influx of extracellular Ca^2+^ through L-type voltage-sensitive channels (VOCs).^17^,^18^

The vasorelaxant effects of graded concentrations of HCE (10–160 mg/ml) were not statistically different (*p* > 0.05) in endothelium-intact aortic rings contracted with low (K^+^, 20 mM) or high (K^+^, 80 mM) potassium concentrations (Fig. 4C).

## Effects of HCE on isolated portal veins

**Fig. 5.**
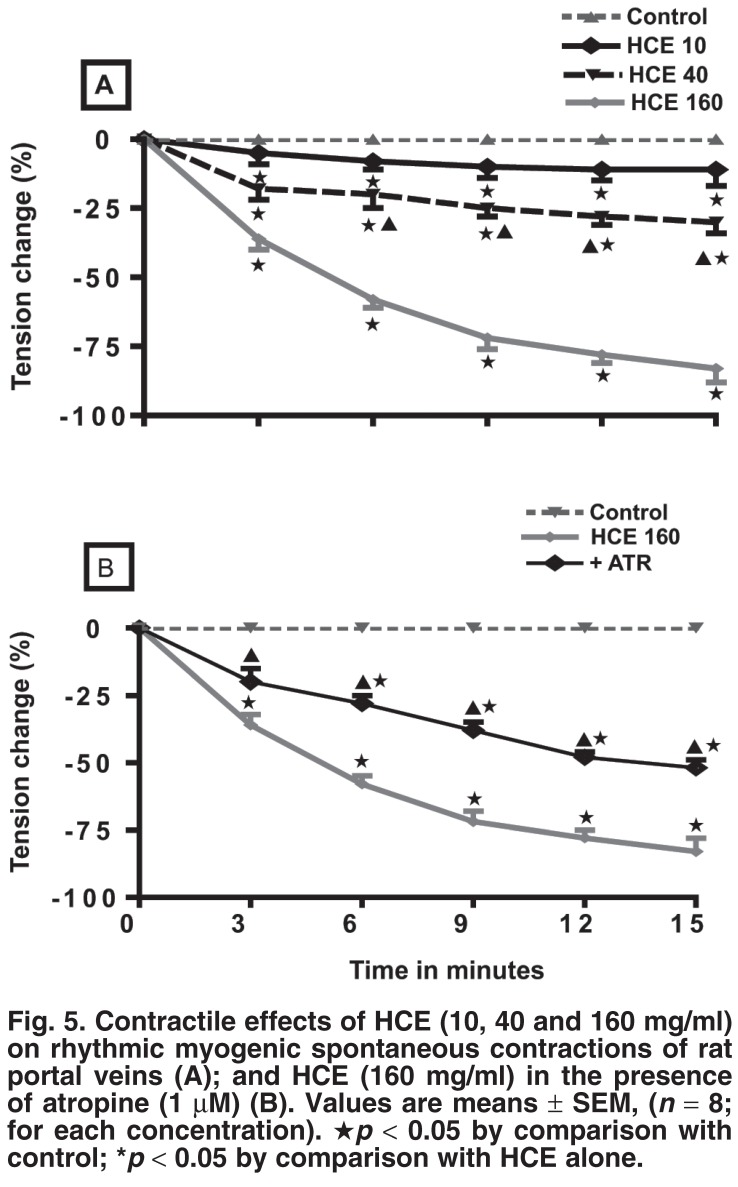
Contractile effects of HCE (10, 40 and 160 mg/ml) on rhythmic myogenic spontaneous contractions of rat portal veins (A); and HCE (160 mg/ml) in the presence of atropine (1 μM) (B). Values are means ± SEM, (*n* = 8; for each concentration). ★*p* < 0.05 by comparison with control; *p < 0.05 by comparison with HCE alone.

The effects of HCE (10, 40 and 160 mg/ml) on the rhythmic, myogenic spontaneous contractions were investigated in rat isolated spontaneously contracting portal veins. HCE induced a significant and concentration-dependent vasorelaxation in these portal vein preparations (Fig. 5A). To investigate if this effect was mediated through cholinergic receptors, the portal vein preparations were pre-treated with atropine (ATR, 1 μM). Pretreatment of the portal vein preparations with atropine, significantly inhibited, but did not abolish the vasorelaxation induced by the highest concentration of HCE (160 mg/ml) (Fig. 5B).

## Discussion

The experimental evidence presented in this study advances our previous report that *Helichrysum ceres* leaf extract reduces blood pressure.^7^ The results indicate that hypotension might be elicited, in part, by direct action on cardiac and vascular smooth muscles. Indeed, HCE elicited potent negative inotropic and chronotropic effects *in vivo* and exhibited vasorelaxant effects in vascular tissue preparations, suggesting that the extract decreases cardiac output and the total peripheral resistance of blood vessels.

Sub-chronic HCE treatment of DSS rats prevented the development of hypertension, presumably via the polyphenols, tannins, triterpenes and saponins present in the extract.^19^-^21^ These compounds have a large variety of pharmacological actions which include stimulation of endothelial nitric oxide (NO) synthesis^22^-^24^ and inhibition of angiotensin converting enzyme (ACE) inhibitory activity.^25^ We suggest that the extract directly or indirectly influenced the cardiovascular and other systems to possibly offer long-term cardioprotection in DSS rats.

Pre-treatment of atrial preparations with atropine completely abolished the negative chronotropic and inotropic effects of lower concentrations of HCE, but partial negative inotropic effects persisted at higher concentrations (160 mg/ml). We speculate that HCE’s cardio-inhibitory effects involve inhibition of β_1_-adrenergic receptors and calcium channels, as evidenced by the extract’s reduction of the noradrenaline or calcium concentration-dependent positive inotropic effects (Fig. 3B). The possibility that HCE at higher concentrations competed with atropine for the cholinergic receptors cannot be excluded.

Removal of the aortic endothelium did not completely abolish the HCE-evoked vasorelaxant effects in rings pre-contracted with ME (α_1_-adrenoceptor agonist) (Fig. 4A), suggesting the involvement of both EDRF-dependent and independent vasodilators. EDRF-dependent involvement was corroborated by the partial inhibition of HCE-elicited vasorelaxations by inhibitors of EDRF: L-NAME for nitric oxide,^26^-^29^ methylene blue for cyclic guanylate cyclase,^30^,^31^ and indomethacin for cyclooxygenase^32^ in endothelium-intact aortic rings pre-contracted with ME. Indomethacin significantly reduced, but did not abolish the vasorelaxant effects of HCE (Fig. 4B), which is also evidence of mediation through the cyclooxygenase pathway.

HCE exhibited concentration-dependent vasorelaxation in endothelium-intact aortic rings pre-contracted with potassium concentrations of 20 mM. Vasorelaxation of aortic preparations pre-contracted with low K^+^ (< 30 mM) occurs via the opening of the potassium channels.^15^,^16^ The vasorelaxant actions of potassium channel-opening agents in smooth muscle tissues are low at high concentrations of K^+^ (> 35 mM).^17^-^18^ The activation of potassium channels and the subsequent membrane hyperpolarisation^33^,^34^ contribute to vasorelaxation.

The vasorelaxant effect of HCE endothelium-intact aortic rings was, however, not significantly affected by pre-treatment with glibenclamide, a selective blocker of K_ATP_ channels.^12^-^14^

We suggest that the vasorelaxant effects of HCE in KCl-contracted aortic rings were mediated by endothelium-derived hyperpolarising factor (EDRF), which directly opens arterial smooth muscle Ca^2+^-activated K_ca_ channels^35^ and relaxes these cells.^36^-^40^ NO-independent vascular vasorelaxation, mediated through the release of endothelium-derived hyperpolarising factor (EDHF), which also opens potassium channels,^41^,^42^ is therefore a possibility in the HCE-elicited vasorelaxation.

HCE elicited concentration-dependent vasorelaxations in the intact aortic rings pre-contracted with high K^+^ (80 mM) concentrations (Fig. 4). The contractile responses induced by high K^+^ in KCl-depolarised muscles are due to the influx of extracellular Ca^2+^ through L-type voltage-sensitive channels (VOCs).^17^ The vasorelaxant effect of HCE against high potassium-induced contraction can be visualised as blockade of Ca^2+^ influx. Taken together, the opening of potassium and calcium channels also appears to be a possible mechanism for the HCE-induced vasorelaxation.

HCE elicited concentration-dependent vasorelaxation in myogenic spontaneously contracting isolated portal vein preparations (Fig. 5A). We suggest that HCE induces this vasorelaxation via stimulation of the cholinergic receptors, since pre-treatment of portal vein preparations with atropine did not completely abolish the vasorelaxation induced by the highest concentration of HCE (160 mg/ml) (Fig. 5B). The vasorelaxant properties of HCE suggest that the extract could provide a source of novel drugs for the management of portal hypertension. Some current pharmacological approaches in the treatment of portal hypertension target reducing portal vascular resistance with vasodilators.^21^

Although the bioactive constituents of HCE were not established in this study, *H ceres* has been reported to contain polyphenols, tannins, triterpenes and saponins with a large variety of pharmacological actions.^22^ Interestingly, these compounds have been reported to stimulate nitric oxide release from vascular endothelial cells to relax blood vessels.^23^-^24^ Studies are currently in progress in our laboratory to isolate the bioactive compounds of this extract.

## Conclusion

The observed vascular effects of HCE provide, in part, not only mechanisms of hypotensive action of the extract, but also the basis for its use in the management of high blood pressure in folkloric medicine.
